# Early Serological Response to BNT162b2 mRNA Vaccine in Healthcare Workers

**DOI:** 10.3390/vaccines9080913

**Published:** 2021-08-16

**Authors:** Giovanna Cocomazzi, Valeria Piazzolla, Maria Maddalena Squillante, Stefano Antinucci, Vincenzo Giambra, Francesco Giuliani, Alberto Maiorana, Nicola Serra, Alessandra Mangia

**Affiliations:** 1 Liver Unit, Fondazione IRCCS “Casa Sollievo della Sofferenza”, 71013 San Giovanni Rotondo (Fg), Italy; g.cocomazzi@operapadrepio.it (G.C.); v.piazzolla@operapadrepio.it (V.P.); m.squillante@operapadrepio.it (M.M.S.); 2 Allergy Diagnostic Section Euroimmun, 35127 Padova, Italy; s.antinucci@euroimmun.it; 3 Institute for Stem Cell Biology, Regenerative Medicine and Innovative Therapies (ISBReMIT) Fondazione “Casa Sollievo della Sofferenza”, 71013 San Giovanni Rotondo (Fg), Italy; v.giambra@operapadrepio.it; 4 ICT Innovation and Research Unit, Fondazione IRCCS “Casa Sollievo della Sofferenza”, 71013 San Giovanni Rotondo (Fg), Italy; f.giuliani@operapadrepio.it; 5 GSSL Unit, Fondazione “Casa Sollievo della Sofferenza”, 71013 San Giovanni Rotondo (Fg), Italy; a.maiorana@operapadrepio.it; 6 Department of Public Health, University “Federico II”, 80131 Naples, Italy; nicola.serra5@gmail.com

**Keywords:** anti-S1, BNT162b2 vaccine, COVID-19 antibody decline, healthcare workers

## Abstract

Purpose: Clinical significance and durability of serological response after mRNA COVID-19 vaccines is under investigation. Data on early virological response are limited. To iden-tify potential predictors of antibody durability, circulating antibody levels were longitudinally ex-plored in healthcare workers included in a follow-up program for SARS-CoV-2 infection. Meth-ods: Subjects meeting the inclusion criteria signed an informed consent. Serum samples were col-lected at baseline, before the first BNT162b2 vaccine, at days 7, 21, 31, 90, and 180 days after the first dose. Serological evaluation was performed by QuantiVac Euroimmune anti-S1 antibody as-say. Only subjects followed-up until day 90 are here considered. Results: Of 340 taken into consid-eration, 265 subjects were naive, and 75 COVID-19 experienced. The former showed a progres-sive increase in their antibody levels before day 90 decline, while the latter showed antibody levels reaching a plateau at day 7 and slightly declining at day 90. All showed antibody levels higher than the assay cut-off at day 31 and 90. Among naive, 108 had an early response whose predic-tors were younger age and female gender (OR 0.94, 95% CI 0.91–0.96, *p* < 0.0001; and OR 2.58, 95% CI 1.48–4.51, *p* = 0.0009). Naive subjects experienced a day 30/90 decline in antibody levels, whereas experienced did not. Early response was an independent predictor of higher day 30/90 antibody levels decline (OR = 2.05, 95% CI 1.04–4.02; *p* = 0.037). Conclusions: Our results suggest that in healthcare workers early response might be inversely associated with antibody levels 90 days after BNT162b2 vaccine.

## 1. Introduction

SARS-CoV-2 virus infection was declared a Public Health Emergency by World Health Organization (WHO) on 30 January 2020 and has currently led up to over 3.72 million deaths globally [1]. The vaccination campaign has started in Europe last 27 December 2020 [2]. However, as of 5 June 2021, in Italy only 21% of individuals have received full vaccination [3]. The BioNTech/Pfizer BNT162b2 mRNA vaccine, the first among others to receive approval [4], showed 95% efficacy and was largely used in healthcare workers [4]. Available data regarding vaccine efficacy are derived by phase III studies on the approved vaccines with established schedules for BNT162b2 mRNA day 1 and day 21 vaccine administration, and by real-world Israeli experience [4,5]. However, shortage of COVID-19 vaccine production and distribution prompted Governments across the world to adopt unique vaccination schedules. More recently, a delay in second dose has been recommended [6,7,8]. Moreover, not univocal indications about timing and schedule were provided for persons who had experienced COVID-19 infection [9,10]. It has been hypothesized that, as adopted in UK for AstraZeneca vaccine, a single dose of BNT162b2 vaccine may be suited to these subjects. This is corroborated by recent studies demonstrating that immune memory involving all four major types of response—i.e. antibodies, memory B cells, memory CD8+ T cells, and memory CD4+ T cells—is measurable in 95% of subjects up to 5 to 8 months post symptoms onset [11,12]. However, information about antibody persistence in individuals previously infected by SARS-CoV-2 are limited [13]. Since neutralizing antibody response is difficult to investigate in large real-life series, kinetics of antibody response and information about its strength and durability after vaccination in persons with or without history of COVID-19 infection might help in orienting future vaccination strategies. Limited data on early humoral response are currently available [14,15]. Consequently, we decided to longitudinally assess the serological response after BNT162b2 vaccine in healthcare workers with or without history of documented COVID-19 infection. The subjects were included in a follow-up program for SARS-CoV-2 infection and in a vaccination campaign started at our Institution on February 2021. Our evaluation started from day 0 before the first dose and continued to at least 180 days after the second vaccine shot.

## 2. Patients and Methods

All healthcare workers enrolled in a prospective hospital vaccination campaign on 3000 subjects were offered to participate into this study starting from 1 February 2021, at IRCCS “Casa Sollievo della Sofferenza” Hospital in San Giovanni Rotondo. Serological evaluation was planned at different time points starting just before the first dose of BioNTech/Pfizer vaccine. Subjects who agreed to participate were divided into three groups: patients with known history of COVID-19 infection, subject without documented COVID-19 infection, but testing antibody positive, and uninfected subjects with both negative history and negative antibodies.

Blood was drawn at baseline, day 0 (d0) (before the first dose), day 7 (d7), day 21 (d21), day 31 (d31) (after the first shot), and day 90 (d90) (60 days after the second shot, respectively). Blood samples were collected into clot activator BD vacutainer tubes (Becton Dickinson, Franklin Lakes, NJ, USA). The margin of sampling window for each timepoint was of 1 day. Individuals for whom a blood sample was missing were excluded from the study.

Semiquantitative serological testing for IgG antibodies anti S1 domain (anti-S1) of SARS-CoV-spike protein was undertaken using the EUROIMMUN anti-SARS-CoV-2 QuantiVac enzyme-linked immunosorbent assay (ELISA) for IgG to the viral spike protein (S-protein) whose positive cutoff was of at least 3.2 BAU/mL. This assay was designed to evaluate vaccine response and calibrated against WHO standard [16]. The cut-off for positivity was 32.5 Binding Arbitrary Unit (BAU), low quantitation limit 3.2 BAU/mL at 1:101 dilution, range (3.2–384.0 BAU/mL). Results ≥25.6 but <35.2 were considered borderline [17]. Specificity and sensitivity (>10 days after diagnosis) are 99.8% and 90.3%, respectively, when the manufacturers’ suggested cutoff ≥35.2 U/mL (or BAU/mL) is used. A solution used for diluting samples above 348 U/mL was included in the measurement kits.

As part of preventive medicine practice, healthcare workers were subjected to routine RT-PCR swab testing using Real Time Reverse transcription PCR kit on a Roche Cobas Z480 thermocycler (Roche Diagnostic, Basel, Switzerland). RNA purification was performed using Roche Magna pure system (Roche Diagnostic, Basel, Switzerland). Both the results of the swab test and the clinical information collected in a dedicated questionnaire were used to confirm previous SARS-CoV-2 infection and were compared to the results of the COVID-19 Regional Registry [18]. Questionnaires were administered in order to ascertain both past symptomatic mild SARS-CoV-2 infection and data regarding family and personal history, in order to identify asymptomatic infections and potential co-morbidities and co-medications.

The results of the swab test performed within the surveillance program were accessed in order to confirm questionnaire answers in patients with recalled SARS-CoV-2 infection. Information about the delay since infections were provided by the COVID-19 experienced participants.

Results attained in patients with past history of COVID-19 positivity were compared at all the different time points with those attained in patients with either no history and baseline positive antibody levels or no history and baseline negative antibody results.

### 2.1. Ethic Approval

All healthcare workers provided written consent in accordance with local review board requirements. Laboratory investigations and available clinical data were collected and analyzed according to the protocol COVIDIAGNOSTIX approved by the EC review board at our institution and funded by Ministry of Health of Italy, “Bando Ricerca COVID-19”; project number: COVID-2020-12371619; project title: COVIDIAGNOSTIX—Health Technology Assessment in COVID serological diagnostics.

### 2.2. Statistical Analysis

Data were presented as number and percentage for categorical variables. Continuous variables were expressed as mean ± standard deviation (SD) or median and interquartile range (IQR). Test for Normal distribution was performed by Shapiro–Wilk test. T-test was used to compare the mean of two independent samples. When the distribution of samples was not normal, T-test with logarithmic transformation was performed. Differences between groups were analyzed using the chi-square test for categorical variables.

Logistic regression was used to find the best fitting model to describe the relationship between the dichotomous characteristic of interest (dependent variable) and a set of independent variables. The variable ratio d31/90 was defined considering the mean of antibody titers ratios at these different time points either in COVID naive or in COVID-experienced subjects.

All tests with *p*-value < 0.05 were considered significant. All data were analyzed by MATLAB statistical toolbox version 2008 (MathWorks, Natick, MA, USA) for 32-bit Windows.

## 3. Results

Overall, 361 subjects provided their informed consent. Twenty-one did not complete the sample collection and were excluded. Of 340 participants analyzed, 195 (57.3%) subjects were female, mean age was 47.7 ± 11.8.

After a careful analysis of the swab test results, 71 (19.6%) COVID-experienced symptomatic subjects were identified. This rate is in keeping with the decision of our Institution to include subjects with a previous COVID-19 infection in the vaccination campaign.

In total, among 269 subjects without recalled past infection, 4 (1.5%) were antibody positive at baseline. These four subjects with contact positive history, but a single negative swab and no previous antibody testing were considered as COVID-19 asymptomatic infections and analyzed together with those with past infections. Consequently, 75 individuals (22.1%) were COVID-19 experienced and 265 naive. Of 75 experienced individuals, 2 (2.6%) had negative antibody despite a positive swab more than 6 and 3 months, respectively, before this evaluation. The first subject was a 65-year-old female taking high doses of steroids for a previous autoimmune disease diagnosis, the second was a 60-year-old woman with asymptomatic COVID-19 infection history and COVID-19 positive family history.

**Figure 1:**
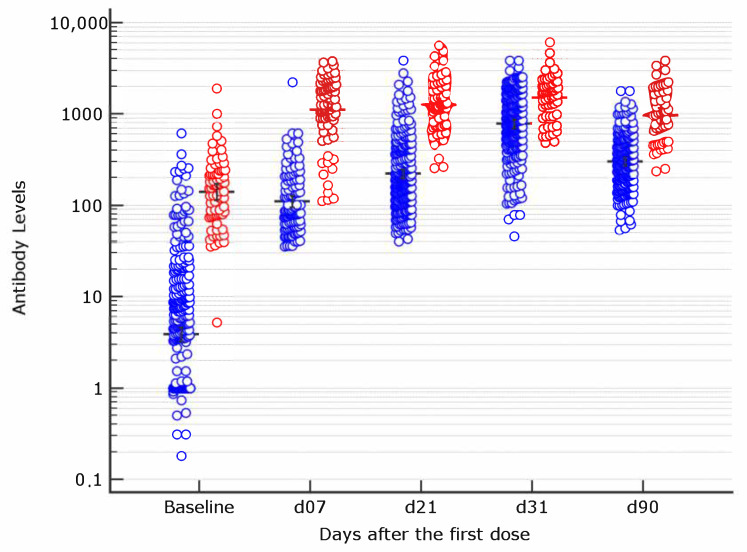
Antibody levels in COVID-19 naive (blue) and COVID-19 experienced (red) at the different time points for subjects with results higher than the positivity threshold of the assay. Geometric mean and 95% Confidence Intervals are reported (statistical significance is shown in Table 1). The highest threshold of the assay is 35,200 BAU/mL.

Overall, antibody titer higher than the assay positivity cut-off 10 days after the second vaccine dose (d31) were observed in 100% of the studied subjects. Sixty days after the second vaccine dose (d90), all had antibody titer above the threshold of positivity (Figure 1).

### 3.1. Patients without COVID-19 Positive Infection History

Of 265 COVID-19 uninfected, 154 (58.1%) were females. Mean age was 48.2 ± 12.5. The proportion of subjects with age ≥ 60 was 16.9%. Among 265 subjects without detectable antibodies at baseline, 108 (40.75%) developed anti-S1 antibodies at a level higher than the assay threshold seven days after the first vaccination dose. These patients were defined as ‘early responders’. This early response was more frequent among female than among male (69.4% vs. 50.3%, *p* = 0.002). Early responders had a mean age of 42.7 as compared to late responders whose mean age was 50.7 years (*p* < 0.0001). The stratification of subjects by age ≥ 60 confirmed the positive association between young age and early response (89.8% vs. 10.2%, *p* = 0.019). Also, the antibody titer decreased by age at this time point, with mean values of 47.7 BAU/mL and 78.50 BAU/mL in older and in young subjects, respectively. Only five subjects were under steroid therapy or other immunosuppressive treatments and this low number prevented the analysis of co-factors potentially associated with early response.

The rate of antibody response increased up to 94.7% at d21 after the first vaccination. Only 14 subjects (7 males and 7 female) did not develop d21 antibody response after the first vaccination dose.

At d31, 10 days after the second dose, all the subjects resulted positive for anti-S1 antibody.

Health care workers were re-tested at d90. At this time point antibody levels declined in almost all. A single subject showed levels of 35.0 BAU which is in the gray zone (≥25.6 but <35.2 BAU/mL) of our assay. Day 7, 21, 31, and 90 antibody response after the first vaccination dose is reported in Table 1

### 3.2. Patients with Previous COVID-19 Positive Infection

Of 75 subjects in this group, 40 (53.3%) were female. Mean age was 47.3 ± 11.6.

At baseline, only two did not develop antibody titers higher than the assay’s threshold of positivity. Among COVID-19 experienced, 11 received a single vaccine dose. The proportion of antibody positive subjects at d7 and d21 is reported in Table 1. Overall, nine subjects (12%) in this group had a baseline titer higher than the highest threshold of our assay as compared to none in the group of uninfected subjects.

As shown in Figure 1, at d7, the COVID-19 positive group showed averaged instrumental signal responses that are approximately 10 times higher than the corresponding COVID-19 negative group. At d21, among patients with previous COVID history, the antibody titer was more than four times higher than the mean titer of 242 subjects testing positive at the same time point among naive. This difference resulted about 2 times at d31 and again 3 times higher, at d90, 60 days after the boosting dose.

The median delay between the first negative swab test after COVID-19 infection and the first vaccination was 90.0 days (IQR 72.5–119.5). Plotting individuals’ time intervals and corresponding antibody levels at the different time points, we failed to observe higher titers by shorter time intervals between first negative swab and first dose vaccination.

The averaged signal observed 21 days after the first dose in COVID-19 positive patients was significantly higher than that observed at day 31, 10 days after the second vaccination dose, in COVID-19 negative patients (*p* = 0.0001).

Eleven of 75 subjects (13.5%) chose not to receive the boosting dose and were separately analyzed from d21 onward. At d31, 10 days after the boosting, only one (1.6%) of the remaining 64 COVID-19 positive patients had an average level lower than the highest threshold of our 1:1000 diluted sample (3520 BAU/mL) as compared to 43 (16.2%) subjects from the group of COVID-19 naive (*p* = 0.002). At d90, all the patients in this group had positive antibodies results; in only three cases, the titer was below the highest threshold of the assay as compared to 149 (58.2%) of those in the group of COVID-19 naive (*p* = 0.001).

The mean ratio between d31/90 levels in this group was 1.83 (95% CI 1.50–2.16).

When antibody titers of COVID-19 experienced subjects at d21 were compared to those of COVID-19 naive subjects at d31, they resulted significantly higher (*p* = 0.0001).

### 3.3. Kinetics of Antibody Response within Each Group

**Figure 2:**
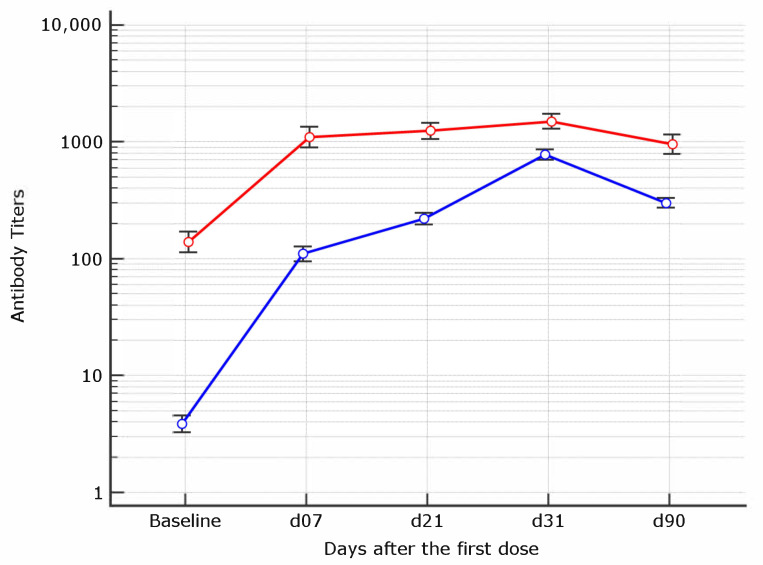
Geometric mean antibody titers kinetics in healthcare workers by prior COVID-19 experience Figure 90 days after the first BNT162b mRNA dose (red line = COVID-19 experienced, blue line = COVID-19 naive). The highest threshold of the assay is 35.200 BAU/mL.

Mean antibody titers and their increment at the different time points were investigated within each group of COVID-19 naive or experienced. The kinetics of antibody response differed between the two groups (Figure 2). Indeed, among naive individuals, not only a mean increment of 18.1 times from baseline to d7, but also an increment of 3–4 times from d7 to d21 and d31 was observed. At d90 all the individuals experienced a decline in antibody titers. By contrast, the viral kinetics of anti-S1 antibody response in COVID19 experienced showed a plateau after d7 and only modest variations thereafter.

### 3.4. Patients Receiving a Single Dose of BioNTech/Pfizer BNT162b2 mRNA Vaccine

Among 11 COVID-19 experienced subjected who decided not to receive the second dose. A single patient got infected after the first vaccine dose and is not included in this analysis. At day 21 after the first vaccine dose, the mean antibody levels of the remaining 10 were 1360.68 ± 1493.65. After 90 days from the first vaccination dose, all remained positive with mean results higher than the highest assay threshold. In no case antibody levels below the assay threshold were observed. The follow up of these subjects is ongoing.

### 3.5. Multivariate Analysis of Factors Associated with Early Vaccine Response in COVID-19 Naive

Independent predictors of early antibody response among COVID-19 naive were investigated by multivariate analysis. The covariates included age and gender.

**Figure 3:**
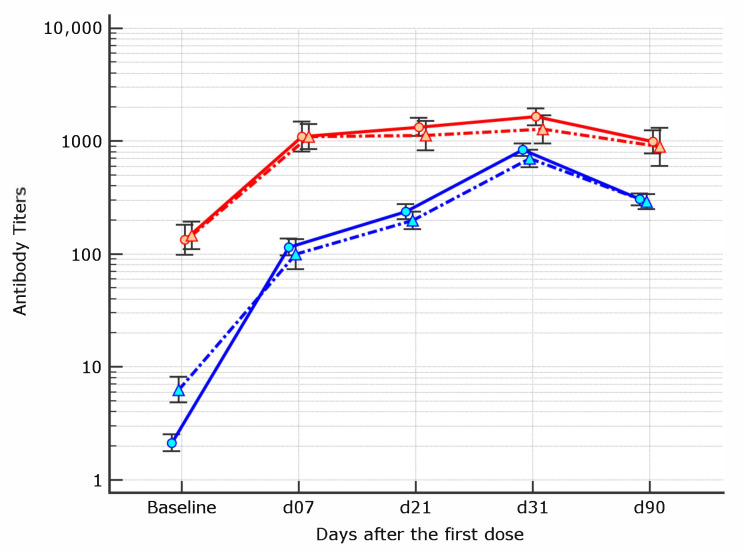
Geometric mean antibody titers kinetics in healthcare workers by prior COVID-19 experience and gender from baseline to 90 days after the first BNT162b mRNA dose (red line = COVID-19 experienced, blue line = COVID-19 naive). The highest threshold of the assay is 35.200 BAU/mL.

Both younger age (OR 0.94, 95% CI 0.91–0.96, *p* < 0.0001) and female gender (OR 2.58, 95% CI 1.48–4.51, *p* = 0.0009) appear to be independent predictors (Figure 3 depicts gender association).

### 3.6. Univariate Analysis of Factors Associated with d31/d90 Antibody Decline in COVID-19 Naive

As the d31/d90 ratio of COVID-19 experienced subjects was 1.83, suggesting a minimal decline in antibody levels, we analyzed all the subjects with a higher ratio threshold amongst COVID-19 naive. Overall, 71.1% of naive subjects showed a decline larger than that observed in COVID-19 experienced. Amongst subjects with low or higher decline, neither male gender (14.2% vs. 15.9%) nor younger age (85.8% vs. 84.1%) was differently distributed. By contrast, a higher proportion of subjects with early response (50.3%) was observed among individuals with more profound antibody level decline in comparison to 31.7% of those with low ratio decline (*p* = 0.016).

### 3.7. Multivariate Analysis of Factors Associated with 90 Days Decline in COVID-19 Naive

Independent predictors of the d31/d90 low ratio were explored (Table 2). Using age, gender and early response as covariates, the only independent predictor of greater decline was an early antibody response (OR = 2.05, 95% CI 1.04–4.02; *p* = 0.037).

## 4. Discussion

In this longitudinal cohort study, we demonstrated that among subjects naive to COVID-19 infection, anti-S1 domain of SARS-CoV-spike protein antibodies are detectable as early as seven days after the first BNT162b mRNA vaccine dose in 40.7% of cases. Subjects who rapidly developed anti-S1 protein antibodies were more often female and young. Interestingly, at d90—60 days after the boosting—a more profound decline in antibodies titer was observed in early responders.

Little is known on the early antibody development and on their quality after mRNA vaccination. A small cohort of 33 healthy SARS-CoV-2 individuals, who received both BNT162b Pfizer and mRNA-1273 Moderna mRNA vaccines and was followed for 7 days after the second dose, has been studied by Goel et al. [14]. The study, that includes memory B cell, neutralizing, anti-S1 and anti-RDB antibody evaluations showed the presence of both anti-S1 and neutralizing antibodies in 50% of subjects as early as 15 days after the first dose [14]. In agreement with our study, this early response was negatively associated—although not significantly—with age [14]. Several studies have suggested that immune senescence play a role after COVID 19 vaccination [19,20]. Negative association between age and anti-S1 protein antibody titers after a single vaccination dose has been reported by others [15,21], although at later timepoints an age-associated lower response remains unclear.

Heterogeneity in antibody response is expected, but the contribution of gender has been debated and possibly related to the sex hormone effect [22], as observed in other health workers cohorts [23]. Whether the higher proportion of female among early responders, regardless of age represents a true difference in the magnitude of single vaccine response will be further investigated.

A novel finding of this study is the possible predictive role of an early humoral response. We acknowledge that it is not yet known if detection of binding anti-SARS-CoV-2 antibodies by commercial assays is associated with protective immunity. Natural infection produces variable antibody quantities and subtypes of different longevity and may induce robust memory B-cell responses, despite low plasma neutralizing activity [24]; however, durability of anti-S1 IgG titers as well as memory B cells, CD8+ T cells and CD4+ T cells need to be longitudinally assessed in vaccinated subjects.

The observed inverse association between early humoral response and a more pronounced d31/d90 decline is corroborated by a study conducted in subjects who received mRNA Moderna vaccine suggesting that not only binding antibodies but also neutralizing antibodies titer decline at six months [25]. The lack of association between d31/d90 decline and advanced age in our study may be the consequence of the age distribution of our population since elderly individuals are underrepresented among healthcare workers.

Durability of antibody response is a largely debated question [13,24]. Studies performed in convalescent patients suggest that immunity might wane, with a decay of neutralizing antibodies response [13]. Although the waning of antibody titers does not necessarily indicate the loss of protection, whether the maintaining of antibodies titers above a certain threshold was required, frequent boosting could be needed in naive individuals [12]. If a progressive decline will be documented during the ongoing follow up it might support the idea of boosting the two vaccination doses with an additional one, very recently advanced by the manufacturers of both mRNA vaccines [26,27]. Whether antibody levels decay is the results of a declining memory B cell response or of a suboptimal T-cell activity [28] is under investigation during an extended follow-up.

Another interesting observation in this study is the tangible difference in viral kinetics and antibody response strength between previously infected and COVID-19 naive healthcare workers. Moreover, the evidence that, amongst subjects with past COVID-19 infection history, averaged signal at d21 was significantly higher than that observed 10 days after the second vaccination dose in COVID-19 naive (Figure 2) suggest—in agreement with others [29]—that a single dose may be sufficient in COVID-19 experienced. Finally, as recently reported, it might prevent the risk of antibody dependent enhancement (ADE) reaction after viral re-exposure [15].

As shown [11,14,24], differences in humoral response to BNT162b2 between COVID-19 naive and experienced individuals are informative on possible vaccination strategies for individuals who have recovered from COVID-19 in terms of schedule and number of doses. Given the existing controversy about whether a second dose should be delayed or completely avoided in order to immunize more people, in light of the current vaccine shortage, our results are in line with immunological studies highlighting distinct antibodies and B cells responses by previous COVID-19 disease history [14]. The Centers for Disease Control and Prevention (CDC) recommend not delaying the second dose of mRNA vaccine longer than 6 weeks [30]. However, in our study a small group of subjects with previous COVID-19 infection history, did not accept the second vaccination dose. Their antibody titers 90 days after the first vaccination resulted comparable to the corresponding levels of COVID-19 experienced who received two vaccine doses.

Limitations of this study include lack of neutralization assay and of exploration of memory B-cell or T-cell immunoresponse. This study includes predominantly health care workers of mean age of approximately 48, individuals aged above 60 years old are less represented. Although these results may not be extended to the general population, the difference observed in humoral response could be amplified in aged individuals. Demonstration of final efficacy of the single dose of BNT162b2 in COVID-19 previously infected subjects is limited in this first report to a 60-day follow-up after the second vaccination dose, although the study is ongoing.

In conclusion, our findings suggest that circulating anti-S1 antibody present as early as seven days after the first mRNA vaccination might possibly help in identifying who will have a more pronounced decline in SARS-CoV-spike protein anti-S1domain antibody levels months after mRNA vaccination. Further immunological studies are ongoing to corroborate our results. Given the worldwide vaccine shortage, our findings suggest that a second dose of BNT162b2 vaccine might not be required in SARS-CoV-2 experienced individuals and might have public health implications.

## Figures and Tables

**Table 1:** Demographic characteristics, percentage of positive and titers of anti-S1 protein at the selected time points by prior COVID-19 experience.

	Prior COVID-19 Experience		
	Yes (*n* = 75)	No (*n* = 265)	*p* Value
Age, mean (SD), y	47.3 (11.6)	48.2 (12.5)	0.31
Sex: Male Female	35 (46.7) 40 (53.3)	111 (41.9) 154 (58.1)	0.59
Baseline SARS-CoV-2-IgG No (%)	73 (97.3)	0	*p* < 0.0001
Baseline SARS-CoV-2-IgG level, Geometric mean, (CI at 95%) Median (IQR)	139.28 (113.28, 171.26) 157 (77.73, 235.76)	2.18 (1.91–2.48) 0.99 (0.99, 5.59)	*p* < 0.0001
Day 7 SARS-CoV-2-IgG Early responders No (%)	75 (100)	108 (40.8)	*p* < 0.0001
Day 7 SARS-CoV-2-IgG level, Geometric mean, (CI at 95%) Median (IQR)	1099.08 (900.05, 1342.14) 1207.66 (871.89–1995.34)	110.43 (95.15, 128.17) 82.28 (60.33–190.14)	*p* < 0.0001
Day 21 SARS-CoV-2-IgG, No (%)	75 (100)	251 (94.7)	*p* = 0.047
Day 21 SARS-CoV-2-IgG level, Geometric mean, (CI at 95%) Median (IQR)	1248.24 (1063.06, 1465.67) 1219.0 (763.57–1977.03)	221.48 (197.32, 248.59) 194.6 (110.18–408.87)	*p* < 0.0001
Day 31 SARS-CoV-2-IgG, No (%)	65 * (100)	265 (100)	*p* = 1
Day 31 SARS-CoV-2-IgG level, Geometric mean, (CI at 95%) Median (IQR)	1500.09 (1290.65, 1743.51) 1706.56 (948.0–2155.0)	781.16 (702.85, 868.20) 851.65 (489.0–1515.8)	*p* < 0.0001
Day 90 SARS-CoV-2-IgG, No (%)	64 * (100)	264 (99.6)	*p* = 1
Day 90 SARS-CoV2-IgG level, Geometric mean, (CI at 95%) Median (IQR)	955.36 (784.77, 1163.02) 972.80 (491.67, 1740.0)	300.75 (274.15, 329.94) 301.91 (182.08, 495.0)	*p* < 0.0001

* 11 patients who received a single vaccine dose, were excluded from day 31 onward. The mean ratio between d31 vs. d90 anti-body levels was 4.73 (95% CI 2.81–6.64).

**Table 2:** Logistic regression analysis of predictors of antibody decline 90 days after the first BNT162b mRNA dose.

Logistic Regression	Coefficient	Standard Error	OR	95% CI	*p*-Value
Null model vs. full model					0.0424 * (C)
Ratio d31/d90 decline /age	−0.02	0.014	0.98	0.96–1.01	0.25
Ratio d31/d90 decline/gender	−0.04	0.32	0.96	0.51–1.81	0.91
ratio d31/d90 Decline/early response	0.72	0.34	2.05	1.04–4.02	0.0372 *

* = significant test; OR = odds ratios; CI = odds ratios confidence interval at 95%; The null model = −2ln(L_0_), where L_0_ was the likelihood of obtaining the observations if the independent variables did not affect the outcome, the full model: −2ln(L_0_), where L_0_ was the likelihood of obtaining the observations with all independent variables incorporated in the model; C = chi-square test.

## Data Availability

Data available on request due to ethical restrictions.
